# Two Year Field Study to Evaluate the Efficacy of *Mamestra brassicae* Nucleopolyhedrovirus Combined with Proteins Derived from *Xestia c-nigrum* Granulovirus

**DOI:** 10.3390/v7031062

**Published:** 2015-03-09

**Authors:** Chie Goto, Shigeyuki Mukawa, Takayuki Mitsunaga

**Affiliations:** 1 NARO Agricultural Research Center, 3-1-1 Kannondai Tsukuba, Ibaraki 305-8666, Japan; E-Mail: aeiou@affrc.go.jp; 2 Life Science Research Institute, Kumiai Chemical Industry Co., Ltd., 3360 Kamo, Kikugawa, Shizuoka 436–0031, Japan; E-Mail: s-mukawa@kumiai-chem.co.jp

**Keywords:** *Mamestra brassicae*, baculovirus, cabbage, broccoli, nucleopolyhedrovirus, granulovirus, enhancement, virus infection, biological control

## Abstract

Japan has only three registered baculovirus biopesticides despite its long history of studies on insect viruses. High production cost is one of the main hindrances for practical use of baculoviruses. Enhancement of insecticidal effect is one possible way to overcome this problem, so there have been many attempts to develop additives for baculoviruses. We found that alkaline soluble proteins of capsules (GVPs) of *Xestia c-nigrum* granulovirus can increase infectivity of some viruses including *Mamestra brassicae* nucleopolyhedrovirus (MabrNPV), and previously reported that MabrNPV mixed with GVPs was highly infectious to three important noctuid pests of vegetables in the following order, *Helicoverpa armigera*, *M. brassicae*, and *Autographa nigrisigna.* In this study, small-plot experiments were performed to assess concentrations of MabrNPV and GVPs at three cabbage fields and a broccoli field for the control of *M. brassicae*. In the first experiment, addition of GVPs (10 µg/mL) to MabrNPV at 10^6^ OBs/mL resulted in a significant increase in NPV infection (from 53% to 66%). In the second experiment, the enhancing effect of GVP on NPV infection was confirmed at 10-times lower concentrations of MabrNPV. In the third and fourth experiments, a 50% reduction in GVPs (from 10 µg/mL to 5 µg/mL) did not result in a lowering of infectivity of the formulations containing MabrNPV at 10^5^ OBs/mL. These results indicate that GVPs are promising additives for virus insecticides.

## 1. Introduction

Japan has a long history of microbial control against insect pests. Kitazima [1] described a disease of *Pieris rapae crucivora*, the first apparent report of granulovirus disease in Japan. In 1959, Koyama compiled a list of epizootic disease pathogens of forest pest insects studied since 1933 [2]. The list covers pathogens of 31 families of economically important forest pests including the cabbage armyworm, *Mamestra brassicae,* a polyphagous pest damaging a wide variety of vegetable, ornamental and forest plants. There were many attempts at microbial control of vegetable insect pests using entomopathogenic viruses since the 1960s. Most of the studies involved characterization of viruses, laboratory bioassays and field evaluations of efficacy, for example, use of nucleopolyhedroviruses (NPVs) against *M. brassicae* [3], *Spodoptera litura* [4,5,6], *Xestia c-nigrum* [7,8], and granuloviruses (GVs) against *Pieris rapae crucivora* [3], *Adoxophyes honmai*, *Homona magnanima* [9], and *Plutella xylostella* [10,11].

At present, three baculovirus formulations are registered for use in Japan. The first formulation was registered in 2003 and consists of a combination of two GVs for control of major tea pests: The smaller tea tortrix, *A. honmai*, and the oriental tea tortrix, *H. magnanima*. The second and third formulations are NPVs developed for *S. litura* registered in 2007 and 2012, respectively*.* The formulation for the two tea tortrix species was developed in the early 1990s and widely used in Kagoshima Prefecture with Ministry of Agriculture, Forestry and Fisheries support. After registration it became popular in other tea-growing districts [12]. On the other hand, the sales of the first registered formulation for *S. litura* were less than 30 kg in 2012 [13], and the other formulation registered in 2012 has not yet been placed on the market.

Slow speed of kill, narrow host range, and high production cost are the main hindrances for practical use of baculoviruses. Genetic modification has been used to improve the insecticidal effect of baculoviruses for more than 20 years. *Autographa californica* multicapsid nucleopolyhedrovirus (AcMNPV) and several other baculoviruses have been successfully engineered by introducing genes of insect-specific toxins and physiological effectors to enhance the speed with which the viruses act on their host [14]. However, in Japan the laws and rules for field use of gene modified microorganisms have not been fully developed and therefore implementation of studies on recombinant baculovirus insecticides is currently considered unfeasible. In this context, selection of viruses with a wider host range and screening for enhancers of insecticidal effect of viruses are possible ways to overcome the problems limiting the use of baculoviruses. From this perspective, *M. brassicae* NPV (MabrNPV) is regarded as a strong candidate, since it has a rather wide host range [15,16] and the potential as a control agent against different pest species. Genome analysis suggested that several NPV isolates originating from larvae of *M. brassicae* in Europe and Asia, an NPV isolated from *Helicoverpa armigera* in China, and an NPV isolate obtained from *M. configurata* (MacoNPV-B) in North America are strains of the same virus rather than different virus species [17]. Our previous study revealed that an NPV strain isolated from *M. brassicae* in Japan (MabrNPV T) was closely related to MacoNPV-B [18]. As for the enhancement of infectivity of NPVs, efforts have been made to find materials to increase virus infectivity. Chemicals, such as phosphatidylcholine [19], boric acid [20,21], and stilbene derived fluorescent brighteners [22,23,24] were reported to have an enhancing effect on NPV infection. However, biological materials such as proteins are more preferable than synthetic chemicals as additives of biological control agents, because they have lower residual properties and are suitable for use in organic crop production. The enhancing effect of a GV isolated from *Pseudaletia unipuncta* was found more than 55 years ago [25]. Similar effects were observed in GVs isolated from *Trichoplusia ni*, *Xestia c-nigrum*, *H. armigera*, and *Spodoptera frugiperda* [26,27,28] as well as in Entomopoxviruses isolated from Lepidopteran insects such as *Pseudaletia separata* and Coleopteran insects such as *Anomara cuprea* (see reviews, [29,30]). The enhancement of GVs originating from proteins, named a synergistic factor, viral enhancing factor, or enhancin, which are components of the capsules of the GVs isolated from *P. unipuncta* and *T. ni* and genes coding the proteins were identified and sequenced [31,32,33,34,35]. Whole genome analysis revealed that *X. c-nigrum* GV (XecnGV) has four homologs of *enhancing* [36,37]. Our previous study confirmed that XecnGV had proteins with potency to improve the efficiency of NPV in their capsules [38]. Our detailed bioassays using an artificial diet or cabbage seedling revealed that a combination of MabrNPV with proteins derived from XecnGV capsules (GVPs) improves the insecticidal activity of MabrNPV against not only *M. brassicae* but also *H. armigera* and *Autographa nigrisigna* [38,39,40,41], suggesting prospects for a new biological insecticide against three important noctuid pests of cruciferous crops. Since the insecticidal activity and persistence of viruses in the field are affected by environmental factors—for example sunlight, rainfall, and temperature—as well as biotic factors such as density and age structure of the pest population, field studies would be necessary to evaluate the efficacy of the combined formulation of MabrNPV with GVPs on the control of these noctuid pests.

For the first step to practical use of the new agent, we performed field plot experiments targeting *M. brassicae* larvae for two years to evaluate efficacy of GVPs as an additive of MabrNPV and to identify interfering issues.

## 2. Materials and Methods

### 2.1. Insects, Viruses and Additives

We used a laboratory culture of *M. brassicae*, *M. brassicae* NPV-T strain (MabrNPV) and *Xestia c-nigrum* GV α-4 (XecnGV). The *M. brassicae* colony was collected in Tsukuba, Ibaraki, Japan, and maintained on an artificial diet (Insecta LFS; Nihon Nosan Kogyo Co., Ltd, Yokohama, Japan) for more than 10 years. The MabrNPV originated from a wild type virus isolate of Dr. Kisaku Akutsu, was propagated in *M. brassicae* larvae. The *X. c-nigrum* GV α-4 (XecnGV) was a genotype obtained by *in vivo* cloning [42] and propagated in *Mythimna separata* larvae maintained on Insecta LFS. Maintenance of insects, preparation of the occlusion bodies (OBs) of MabrNPV and capsules of XecnGV were performed as described previously [38]. Alkaline soluble components of XecnGV capsules (GVPs) were prepared from dried capsules, following dissolution in 20 mM NaOH solution, neutralization with HCl, and ultra-centrifugation for separation from virions of XecnGV and undissolved substances [38]. Concentration of GVPs was assessed from the weight of dried capsules.

### 2.2. Field Trials

Field trials were conducted at NARO Agricultural Research Center, Tsukuba, Ibaraki, Japan. Four independent field trials were performed and a completely randomized design was used for each trial with three replicates of each treatment combination.

The same methods were used for the general organization of field trials. Three to four leaf stage cabbage (variety, Kinkei 201) or broccoli (variety, Haitsu SP) seedlings depending on the experiment were planted using a 70 cm × 50 cm grid. Before transplanting, seedlings in nursery boxes were treated with Acetamiprid (2% granule) at 0.5 g/plant to prevent infestation of aphids. In some cases, plants were treated with Bt spray (*Bacillus thuringiensis* subspecies kurstaki, OAT Agrio Co., Ltd., Tokyo, Japan) for the control of white butterfly at a period without any adverse effects on the experiment. Four to five weeks after planting, nine plots with 6 × 5 plants (10.5 m^2^ per plot) were arranged in the field, and nearly hatching or hatching egg masses of a laboratory culture of *M. brassicae* were introduced to seven or eight plants around the center of each plot. At the introduction, small pieces of paper with egg masses were stapled on the back of leaves at the middle of each plant and newly hatched larvae were sprinkled on the leaves. About a week after hatching of the larvae, trial formulations were applied using a 2-head nozzle 5-liter handheld pressure sprayer (Koshin Ltd., Kyoto, Japan) at an application spray volume of 200 L/10 a.

Control plots received a water spray containing only the additives, skimmed milk powder (0.35% w/v), white carbon (Tokuseal NP, Tokuyama Co., Ltd., Osaka, Japan, 0.2% w/v), and a wetting agent (Gramin S, Mitsui Chemicals Agro, Inc.; Tokyo, Japan, 0.03% v/v). MabrNPV was applied at 1 × 10^5^ or 1 × 10^6^ OBs/mL, and GVPs were applied at 0, 5 or 10 µg/mL combined with OBs of MabrNPV. Details of the trials are summarized in Table 1.

**Table 1:** Details of the field trials in 2008 and 2009.

	First trial, 2008	Second trial, 2008	First trial, 2009	Second trial, 2009
Plant	cabbage	cabbage	cabbage	broccoli
Date of transplanting	15 4 2008	23 5 2008	20 4 2009	12 8 2009
Bt treatment	Bt kurstaki 15 5 2008	None	None	Bt kurstaki 3 9 2009
Date of pest release	27 5 2008	29 6–1 7 2008	29–30 5 2009	11–12 9 2009
(quantity/plant)	(*ca.* 160 eggs)	(*ca.* 320 eggs)	(300–400 eggs)	(300–400 eggs or larvae)
Date of virus application	2 6 2008	7 7 2008	4 6 2009	19 9 2009
(Larval stage)	(late 1st-instar)	(2nd to 3rd-instars)	(2nd-instar)	(2nd-instar)
Concentration of NPV	10^6^ OBs/mL	10^5^ OBs/mL	10^5^ OBs/mL	10^5^ OBs/mL
Concentration of GVPs	0 or 10 μg/mL	0 or 10 μg/mL	5 or 10 μg/mL	5 or 10 μg/mL
Date of larval collection	6 6 2008	11 7 2008	8 6 2009	23 9 2009
(Larval stage)	(late 2nd)	(4th)	(late 2nd to mid-3rd)	(3rd to 4th)

In the first 2008 trials, counts of live larvae were performed on May 30 (three days after egg inoculation) by surveying plants which were given inoculation of *M. brassicae*. Collections of larvae were performed at four days after virus treatment by harvesting one plant from each plot, and all the larvae were reared individually as described below.

In the second 2008 trial, the larval collection was performed at four days after virus application harvesting one plant from each plot.

In the 2009 trials, the experimental fields were prepared in the same way as the 2008 trials. Nested design was used for checking the unevenness of the application, and three plants were harvested from each plot (“plant” as nested factor). From each plant, 36 larvae were collected and reared individually.

In the second 2009 trial, fields were prepared in the same way as the previous trial except broccoli was used instead of cabbage.

### 2.3. Rearing of Larvae and Examination of the Cause of Death

Field collected larvae were reared individually in a plastic 1/2 ounce cup on Insecta LFS at 25 °C under 16 L 8 D. Larvae were observed daily for mortality until 13 or 14 days after collection. Larvae with typical symptoms of NPV infection, such as a waxy appearance and liquefaction of the cadaver, were recorded as being infected. Tissue smears were prepared from larvae that had died without symptoms of NPV infection or emergence of parasitoid to test for the presence of OBs under a phase-contrast microscope. Larvae that died from handling were excluded from the following analysis.

### 2.4. Data Analysis

All statistical analyses were performed using JMP software version 9 (SAS Institute, Inc., Cary, NC, USA). Longevity data were tested using Cox’s proportional hazard analysis (CPHA) [43] to compare the hazard ratios between all possible pairwise treatments on survival. To estimate median lethal times (LT_50_s) required for the events such as death caused by the NPV-infection or emergence of parasitoid larvae from host, mortality data after excluding cases of censored data were analyzed by means of a parametric survival analysis approximated by log-normal distribution [44]. For post-hoc comparisons between two specific LT_50_s, a log-rank test weighted by Bonferroni was used to correct for experiment-wide error rate, α [45]. This resulted in a corrected α for the treatment comparison (α = 0.05/*k*, *k* = 3 for all treatment combinations).

## 3. Results

### 3.1. Field Trial in 2008

In the first trial, the survey of live larvae on plants in each plot was performed on May 30 (three days after inoculation of egg masses). At the first survey, hatching and settlement rates of the inoculated *M. brassicae* were very high, un-hatched eggs were observed only in one of 72 egg masses, and failure of colonization of the larvae was found only on a few plants followed by predators such as web-forming spiders. The virus formulations were applied in the afternoon on June 2 when most of the larvae were in the first moulting stage.

**Figure 1:**
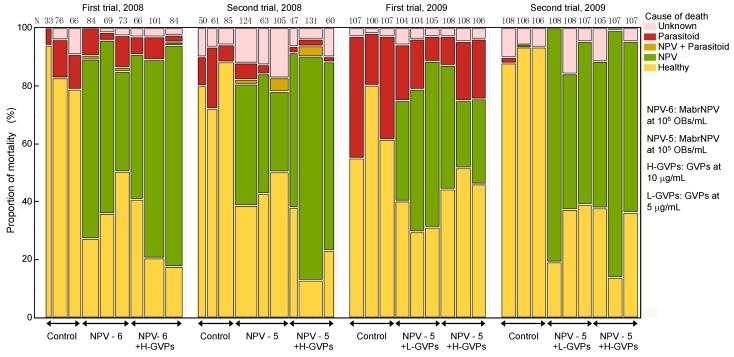
Proportion of mortality caused by NPV infection and parasitoids in the 2008 and 2009 field trials. The number above each column represents the number of larvae for each treatment plot by trial.

Proportions of mortality caused by NPV-infection and parasitization in larvae collected from field plots at four days post-application are shown in Figure 1. No virus mortality was observed in larvae collected from the control plots.

The CPHA was performed to evaluate the effect of virus applications on the mortality of larvae. In this analysis, larvae distinguished to be in the fifth or sixth instars at the collection were eliminated from the data, because they were assumed to be less susceptible to NPV than larvae originated from inoculated egg masses. However, some of them from plots applied with formulations containing MabrNPV died with NPV infection. The CPHA indicated that the application of the formulations of MabrNPV either mixed with or without GVPs caused a significant increase in larval mortality (Table 2). The hazard ratios of MabrNPV alone/Control, MabrNPV+GVPs/Control, and MabrNPV + GVPs/MabrNPV alone were 5.20, 7.35, and 1.41, respectively (Table 3). Furthermore, there was a small but significant difference in LT_50_s of larvae infected with NPV between the treatments with NPV alone and NPV with GVPs (Table 4).

Parasitoid larvae, mainly larvae of solitary braconid wasps, emerged from around 10% of *M. brassicae* larvae regardless of the treatments, and the survival analysis indicated that there was no significant difference in the mortality of larvae caused by parasitization among the three treatments (Table 2). Most of *M. brassicae* larvae survived for a week or longer after the emergence of parasitoid larvae. A few of the host larvae died with typical symptoms of NPV infection and production of OBs was confirmed by microscopic examination of the cadavers.

At the collection on June 6, a small number of *A. nigrisigna* larvae were captured and reared with the larvae of *M. brassicae*. A larva collected from a control plot pupated, three of four larvae collected from a plot treated with MabrNPV alone and one of two larvae collected from a plot treated with MabrNPV combined with GVPs died with NPV infection.

**Table 2:** Cox’s proportional hazard analysis (CPHA) of larvae collected from fields at four days post-application of virus formulations.

Factor	Overall analysis ^2^			Viral Infection ^3^			Wasp Emergence ^4^		
	*df*	*G*	*p*	*df*	*G*	*p*	*df*	*G*	*p*
First trial, 2008 (Cabbage)									
Treatment	2	153.24	<0.01	1	9.62	<0.01	2	1.83	0.400
Second trial, 2008 (Cabbage)									
Treatment	2	146.95	<0.01	1	50.94	<0.01	2	6.57	0.038
First trial, 2009 (Cabbage)									
Treatment	2	75.63	<0.01	1	13.79	<0.01	2	4.67	0.097
Plant ^1^	18	47.88	<0.01	12	35.08	<0.01	18	35.19	<0.01
Second trial, 2009 (Broccoli)									
Treatment	2	340.21	<0.01	1	1.76	0.185		- ^5^	
Plant ^1^	18	102.39	<0.01	12	74.18	<0.01			

^1^ The effect was analyzed by using a nested model with a multi-level structure, treatment > replication > plants; ^2^ Larvae surviving at the end of the observation period were treated as censored data; ^3^ Dead larvae without NPV infection or surviving larvae at the end of the observation period were treated as censored case; ^4^ Dead larvae without parasitization or surviving larvae at the end of the observation period were treated as censored data; ^5^ Parasitization was observed in only 2 larvae collected from the control plots in this trial.

**Table 3:** The hazard ratios estimated by the CPHA of larvae collected from fields at four days post-application of virus formulations.

	Overall Mortality			Mortality by Virus			Mortality by Wasps		
Treatment (crop)	Hazard Ratio ^1^	95% CL		Hazard Ratio ^1^	95% CL		Hazard Ratio ^1^	95% CL	
Pairwise Treatment		Lower	Upper		Lower	Upper		Lower	Upper
First trial, 2008 (Cabbage)^2^									
NPV alone/Cont.	5.20 *	3.54	7.90				1.51	0.81	2.81
NPV + GVPs/Cont.	7.35 *	5.04	11.11				1.37	0.69	2.66
NPV + GVPs/NPV alone	1.41 *	1.14	1.76	1.44 *	1.14	1.83	0.91	0.46	1.76
Second trial, 2008 (Cabbage)^3^									
NPV alone/Cont.	3.57 *	2.53	5.18				0.51 *	0.26	0.96
NPV + GVPs/Cont.	6.47 *	4.59	9.36				0.43 *	0.19	0.88
NPV + GVPs/NPV alone	1.81 *	1.47	2.24	2.37 *	1.87	3.02	0.84	0.37	1.84
First trial, 2009 (Cabbage)^4^									
Low GVPs/Cont.	2.77 *	2.19	3.54				0.83	0.58	1.18
High GVPs/Cont.	1.90 *	1.49	2.45				0.69 *	0.48	0.97
High GVPs/Low GVPs	0.69 *	0.56	0.84	0.61 *	0.46	0.79	0.82	0.55	1.23
Second trial, 2009 (Broccoli)^4^									
Low GVPs/Cont.	17.37 *	10.37	34.53						
High GVPs/Cont.	19.79 *	11.81	39.33						
High GVPs/ Low GVPs	1.14	0.94	1.38	1.15	0.94	1.40			

^1^ The values with asterisks are significantly different from 1. ^2^ NPV alone: 10^6^ OBs/mL of MabrNPV; NPV + GVPs: 10^6^ OBs/mL of MabrNPV + 10 μg/mL of GVPs. ^3^ NPV alone: 10^5^ OBs/mL of MabrNPV; NPV + GVPs: 10^5^ OBs/mL of MabrNPV + 10 μg/mL of GVPs. ^4^ Low GVPs: 10^5^ OBs/mL of MabrNPV + 5 μg/mL of GVPs ; High GVPs: 10^5^ OBs/mL of MabrNPV + 10 μg/mL of GVPs.

**Table 4:** Median lethal times of *Mamestra brassicae* larvae collected from field plots treated with MabrNPV.

Treatment (Crop)	No. of Observation	LT_50_ (Days)	95% CL		Parametic Survival Analysis		
Ingredients of Formulations			Lower	Upper	*df*	*G*	*p*
First trial, 2008 (Cabbage)							
10^6^ OBs/mL of MabrNPV alone	122	5.0	4.8	5.2	1	4.91	0.027
10^6^ OBs/mL of MabrNPV + 10 μg/mL of GVPs	167	4.7	4.6	4.9			
Second trial, 2008 (Cabbage)							
10^5^ OBs/mL of MabrNPV alone	113	4.2	3.9	4.5	1	5.87	0.015
10^5^ OBs/mL of MabrNPV + 10 μg/mL of GVPs	169	3.7	3.5	4.0			
First trial, 2009 (Cabbage)							
10^5^ OBs/mL of MabrNPV + 5 μg/mL of GVPs	147	4.2	4.0	4.5	1	0.55	0.459
10^5^ OBs/mL of MabrNPV + 10 μg/mL of GVPs	102	4.4	4.1	4.7			
Second trial, 2009 (Broccoli)							
10^5^ OBs/mL of MabrNPV + 5 μg/mL of GVPs	197	4.2	4.0	4.3	1	1.77	0.184
10^5^ OBs/mL of MabrNPV + 10 μg/mL of GVPs	207	4.0	3.9	4.2			

^1^ Median lethal times were determined by using the parametric survival analysis approximated by log-normal distribution after excluding the data of censored case.

In the field, larvae with symptoms of NPV infection, such as sluggish movement at the upper area of leaves, whitish body color, and leaking of body fluid, were observed at least nine days post-application.

In the second 2008 trial, the concentration of OBs of MabrNPV in the formulations was adjusted to 1 × 10^5^ OBs/mL, equivalent to 1/10 of that in the first trial. Proportions of larval mortality caused by NPV-infection and parasitization are shown in Figure 1. No virus mortality was observed in larvae collected from the control field. Overall analysis of the CPHA confirmed that there were significant differences in larval mortalities among the three treatments (Table 2), and significant hazard ratios of NPV alone/Control, NPV + GVPs/Control, and NPV + GVPs/NPV alone were detected as 3.57, 6.47, and 1.81, respectively (Table 3). The re-analysis of the data excluding the control treatment revealed that addition of GVPs caused a significant increase in NPV infection (Table 2), and the infection risk of the NPV + GVPs treatment was 2.37 fold higher than that of the NPV alone treatment (Table 3). There was a significant difference in LT_50_s of the NPV infected larvae between the treatments with NPV alone and NPV combined with GVPs (Table 4). The proportion of parasitized larvae collected from control plots was almost the same as that in the first trial, while those of the plots applied with formulations containing MabrNPV were lower than those in the first trial (Figure 1). The CPHA indicated that the mortality of larvae caused by parasitization was different among the three treatments (Table 2). The hazard ratios which were significantly lower than 1 in NPV alone/Control and NPV + GVPs/Control indicated that the NPV treatments influenced negatively on parasitism (Table 3). However, parametric survival analysis did not detect any difference in time period for parasitoid emergence by treatments (Table 5).

**Table 5:** Times required for 50% emergence of parasitoid larvae from hosts.

Treatment (Crop)	No. of Observation ^1^	50% Emergence (Days) ^2^	95% CL		Parametric Survival Analysis		
Ingredients of Formulations			Lower	Upper	*df*	*G*	*p*
Second trial, 2008 (Cabbage)							
Control	23	3.1 a	2.47	3.90			
10^5^ OBs/mL of MabrNPV alone	16	2.1 a	1.56	2.70	2	5.06	0.080
10^5^ OBs/mL of MabrNPV + 10 μg/mL of GVPs	10	2.4 a	1.72	3.43			
First trial, 2009 (Cabbage)							
Control	102	6.2 a ^3^	5.9	6.4			
10^5^ OBs/mL of MabrNPV + 5 μg/mL of GVPs	50	6.5 ab	6.2	6.7	2	12.37	0.002
10^5^ OBs/mL of MabrNPV + 10 μg/mL of GVPs	56	6.9 b	6.6	7.3			

^1^ Survival analysis was performed based on the data of dead larvae due to wasp parasitization; ^2^ Median emergence times were determined by using the parametric survival analysis approximated by log-normal distribution after excluding the data of censored case; ^3^ The 50% emergence times with the same letters are not significantly different from each other. (Log-rank test with Bonferroni correction (*p*<0.05/3)).

In the field, larvae with symptoms of NPV infection were observed at nine days post-application. At the collection of larvae on July 11, a small numbers of *A. nigrisigna* larvae were captured and reared in the same way as *M. brassicae* larvae. One of 16 larvae collected from plots treated with MabrNPV alone, and four of 13 larvae collected from plots treated with MabrNPV combined with GVPs died with NPV infection. None of the 12 larvae collected from control plots died with NPV infection.

### 3.2. Field Trial in 2009

The experimental fields were prepared in the same way as the 2008 trials. In the first 2009 trial, the egg masses were put on seven plants around the center of each plot, from May 29–30. The virus formulations were applied in the afternoon on June 4 when most of the larvae were in the second instar. At four days post-application, when larvae were in late second to third instars, collection of larvae was performed and 36 larvae from each of the three plants per plot were reared individually in the laboratory. Proportions of mortality caused by NPV infection and parasitization of larvae are shown in Figure 1. The mean mortality in the Low-GVPs treatment was higher than that in the High-GVPs treatment, contrary to expectations. The overall mortality, the mortality caused by NPV infection, and the mortality caused by parasitization were analyzed using the CPHA. The survival times of all larvae and larvae infected with NPV were affected by both “treatment” and “plant” and that of larvae parasitized by wasps was affected by “plant” (Table 2). Re-analysis of the mortality data caused by the NPV infection (excluding the control treatment) showed a significant difference between the Low- and High-GVPs treatments (Table 2). Hazard ratio indicates that the infection risk in the Low-GVPs treatment was higher than that in the High-GVPs treatment in this trial (Table 3). However, there was no significant difference between the LT_50_s of the NPV infected larvae in the two treatments (Table 4).

Mean parasitization rates of larvae in the control, the High-GVPs treatment, and the Low-GVPs treatment were 31.9% (102/320), 18.6% (56/322), and 15.9% (50/313), respectively. There was no significant difference among mortality caused by parasitized larvae in the three treatments (Table 2), though the significant hazard ratio showed that the risk of parasitism in the control treatment was higher than that in the High-GVPs treatment (Table 3). Re-analysis of the data was conducted omitting the effect of plant (Table 5). Log-rank tests between pairs of three treatments indicated that time required for the emergence of the parasitoid larvae in the control treatment was not significantly different from that in the Low-GVPs treatment, but significantly shorter than that in the High-GVPs treatment (df = 1, *G* = 8.048, *p* = 0.0046). Time required for the emergence of the parasitoid larvae in the High-GVPs treatment was not significantly different from that in the Low-GVPs treatment. An additional parametric survival analysis indicated that there was a significant difference in survival distributions of parasitized larvae between control and combination of the High- and the Low-GVPs treatments (df = 1, *G* = 9.313, *p* = 0.0023), and times required for the 50% emergence of the parasitoid larvae were estimated to be 6.2 days (95% CL: 5.9–6.4 days) in the control and 6.7 days (95% CL: 6.5–6.9 days) in the combined treatments with NPV.

There were only two dead larvae with fungal infection in this trial, and these were treated as dead with “Unknown” cause of death in Figure 1. At eight days post-application and after, larvae with typical symptoms of NPV infection were observed in the field.

In the second 2009 trial, the experiment was performed in the same way as the previous trial except broccoli was used instead of cabbage. The egg masses and newly hatched larvae were put on eight plants around the center of each plot, from September 11–12. The virus formulations were applied in the afternoon of September 19, when most of the larvae were in the mid to late second instar. At four days post-application, larvae in late third to fourth instars were collected from three plants in each plot, and 36 larvae from each plant were reared individually in the laboratory. Proportions of mortality caused by the NPV-infection and parasitization in larvae are shown in Figure 1. In this trial, parasitization by wasps was observed in only two larvae collected from the control plots, suggesting very low parasitization rate of larvae.

The overall mortality and the mortality caused by the NPV infection were analyzed using the CPHA. The survivorship of all larvae was affected by both “treatment” and “plant”, and re-analysis of the mortality data caused by the NPV infection (excluding the control treatment) revealed that there was no significant difference between survivorship of larvae in the Low- and the High-GVPs treatments (Table 2). The insignificant hazard ratio in High-GVPs/Low-GVPs also indicates that the concentration of GVPs did not affect the viral infection rate, while over 15-fold higher mortality risk in both Low- and High-GVPs treatments (17.37 and 19.79, respectively) was shown against that in the control treatment (Table 3). There was no significant difference between the LT_50_s of the NPV infected larvae in the two treatments (Table 4).

At nine days and after post-application, dead or near death larvae with typical symptoms of NPV infection were observed on broccoli plants in the experiment fields.

## 4. Discussion

Baculoviruses are considered attractive alternatives to broad-spectrum insecticides because of their specificity, and their lack of untoward effects on beneficial insects makes them ideal components of IPM systems. In other words, the adoption of a sound IPM program by farmers is indispensable for wide use of viral insecticides. The successful penetration of baculovirus insecticides in IPM systems depends on a combination of factors including selection of the most virulent isolates [46,47]. High selectivity of baculoviruses is a favorable characteristic in regards to environmental safety, but it has often deterred commercial development due to limited market size [48]. MabrNPV is one of the promising insect pathogens, not only because the original host is a very important pest in vegetable and ornamental plant production but also because it can infect many important noctuid pests [15,16]. Additional demerits of the use of baculoviruses are their relatively slow action and high costs for *in vivo* production. Since the 1990s, many attempts were performed to improve the insecticidal activity of NPVs by introducing genes of insect-specific toxins or genes related to hormone regulation into genomes of NPVs and some of them showed high performance under field conditions comparable to that of chemical insecticides [14]. Nevertheless, there have been insurmountable obstacles over the field use of gene modified viruses in many countries including Japan.

The other approach is searching for substances to improve insecticidal activity of baculoviruses. As for chemicals, the ability of boric acid to enhance virus infectivity was successfully demonstrated not only in laboratory [20] but also in field and semi-field studies [21]. Stilbene-derived optical brighteners were reported to act as absorbents of ultraviolet radiation and as potent synergists of NPVs including MabrNPV [22,23,38,49]. Application of *Spodoptera exigua* MNPV in mixtures with a brightener, leucophor AP resulted in a significant increase in the prevalence of infection in larvae of *S. exigua* in laboratory studies. In greenhouse sweet pepper crops, the effect of leucophor AP was identified in larvae collected at two days post-application, but not in insects collected subsequently [24]. While field studies carried out in Mexico and the United Kingdom indicated that application of dilute concentrations of the optical brightener, Tinopal CBS, reduced recruitment of bees to flowers [50]. This report suggested that more detailed studies of possible undesirable side effects of synergist candidates, especially stable chemicals, should be conducted for successful development of viral pesticides. As for pesticides, Flufenoxuron, an insect growth regulator (IGR) [51], and two chitin synthesis inhibiting antifungal agents named Captan and Polyoxin AF [52], were reported to increase NPV susceptibility of the silkworm larvae *Bombyx mori* when incorporated into the insect’s artificial diet as enhancers of the NPV. However, fungicides containing polyoxin-d failed to increase *Agrotis ipsilon* MNPV (AgipMNPV) infectivity to *A. ipsilon* in a turf grass field [53]. In addition, no beneficial effect attributable to the inclusion of a brightener, M2R, in AgipMNPV formulations was detected under greenhouse or field conditions, though M2R showed high activity of enhancement on the NPV infection in laboratory droplet feeding bioassays [54].

Biological substances such as proteins produced by entomopoxviruses [30] and granuloviruses [25,26,27,28,55] are also known as synergists of NPVs. Although there have been many laboratory studies on the enhancing activities of these proteins, there is little information on the field efficacy of these substances.

Laboratory bioassays can be considered a type of forced feeding. On the contrary, there might be many factors impeding infection, especially feeding, correlated with curtailment of synergistic effects of selected substances in field experiments. From this point of view, we should carefully evaluate the synergistic effect of proteins derived from XecnGV capsules.

Our previous study with detailed bioassays using an artificial diet and cabbage plants show that a combination of MabrNPV and GVPs has the potential for simultaneous control of *M. brassicae*, *H. armigera*, and *A. nigrisigna* [38,39,40]. As described above, there would be a certain degree of difference between substances with synergistic activity of NPVs in laboratory and field experiments.

For the first step of practical use of MabrNPV combined with GVPs, we performed field experiments at cabbage plots with high densities of larvae prepared by inoculation of mature eggs of *M. brassicae* in 2008. The tentative formulation containing MabrNPV at 10^6^ OBs/mL resulted in a 63% mean larval mortality (53% lethal NPV infection) and addition of GVPs (10 µg/mL) to the formulation caused a significant increase in infectivity (66% lethal NPV infection). The CPHA indicated that the hazard ratio of MabrNPV + GVPs/NPV alone was about 1.4 (Table 3). The insecticidal activity of the formulation was lower than that expected from the results of our laboratory studies; however, the enhancing effect of GVPs was confirmed for the first time under field conditions. In addition, LT_50_ of NPV infected larvae collected from plots treated with NPV combined with GVPs was significantly shorter than that of larvae collected from plots treated with NPV alone (Table 4). In the second trial, the mean NPV-infection rate at the plots applied with the formulation containing MabrNPV at 10^5^ OBs/mL was less than 50%, while, addition of GVPs resulted in significantly increasing the mean NPV-infection rates and the formulation containing MabrNPV and GVPs considered to have comparable insecticidal effect to that with 10-times higher concentrations of MabrNPV alone in the first trial. The hazard ratios of MabrNPV + GVPs/NPV alone for overall mortality was about 1.8, and that for mortality by virus infection was 2.4, therefore, both were larger than those in the first trial (Table 3). This indicates that there was not much room for improvement in MabrNPV infection by addition of GVPs when MabrNPV was applied at 10^6^ OBs/mL, and a 10-time reduction in MabrNPV concentration made the effect of GVPs more striking. Similar to the first trial, the addition of GVPs significantly reduced the LT_50_ of NPV infected larvae (Table 4). Our previous bioassays using the second instar larvae of *M. brassicae* [38], *H. armigera* [39,40] and *A. nigrisigna* [41] indicated the effect of GVPs not only increases the infectivity of MabrNPV but also shortens the lethal time and/or causes death at a younger instar. These trials in 2008 verified the desirable effects of GVPs on MabrNPV infection in the cabbage field. In addition, *A. nigrisigna* larvae collected from the NPV-treated plots with *M. brassicae* larvae, died with NPV infection during rearing. These results agreed with our previous reports of bioassays, indicating a wide host range of MabrNPV.

In the 2008 trials, larvae were collected from only one plant at each plot, so it was difficult to evaluate unevenness in the insecticidal effect of formulations, though there seemed to be considerable differences in proportions of mortality among plots (Figure 1). Baculoviruses must be ingested to be effective and variation in distribution of OBs on the surface of crops may cause a reduction in insecticidal activity of formulations. Therefore, in the 2009 trials, a nested design was conducted and larvae were collected from three plants at each plot to evaluate the effect of plant on difference in viral mortality among plants. Moreover, broccoli, with leaves that are oriented more vertically than those of cabbage, was used in the second trial instead of cabbage. We also applied an NPV formulation with reduced amount of GVPs instead of the NPV alone formulation to check the possibility of reducing the production cost without diminishing insecticidal activity of the formulation. In the first 2009 trial, against our expectation, mortality caused by viral infection in the treatment with MabrNPV combined with GVPs at 5 µg/mL was significantly higher than that in the treatment with MabrNPV combined with twice as much GVPs. On the other hand, there was no significant difference between LT_50_s of the virus infected larvae in these two treatments. In the second 2009 trial, there were no significant differences in mortality and LT_50_s of the virus infected larvae between the two treatments. These results indicated that reducing the concentration of GVPs by half cause no adverse effect on the infectivity of the MabrNPV, while, the factor “plant” had a significant effect on the virus infection. In the field, larvae tended to remain on the reverse side of the leaves where the egg mass was attached during their first and second stages and gradually dispersed as they grew. It seemed difficult for the manual application method to distribute the formulation evenly and adequately cover both sides of all leaves, and this could be one of the reasons for the level of larval mortality in the plot with MabrNPV combined with GVPs at 10 μg/mL in the first trial in 2009. Cabbage leaves tend to fold over from the edges as they grow and overlap each other more than broccoli, so we expected that the effect of plant might be less in broccoli than in cabbage, but effects of plant were significant in both trials (Table 2). In these trials, application of NPV formulations apparently reduced the damage of plant leaves; however, large numbers of larvae remained behind. It was natural that only one application of the NPV formulation could not control the artificial outbreak of *M. brassicae*. More detailed studies concerning the optimum timing, frequency, and application methods are necessary for the practical use of the formulations containing MabrNPV and GVPs.

The mortality of larvae collected from control plots four days post-application rarely exceeded 20% except in the first 2009 trial (Figure 1), and the main cause of death was parasitization by wasps in all trials. In the second trial in 2008, a significant difference was detected with the CPHA in the mortality caused by parasitization among the three treatments (Table 2), and the estimated hazard ratios indicated that the application of MabrNPV caused significant reduction of parasitization (Table 3). The additional survival analysis indicated that time required for the emergence of the parasitoid larvae in the control treatment was not significantly different from that in the Low-GVPs treatment, but significantly shorter than that in the High-GVPs treatment. These results suggest that there are some interactions between parasitoids and MabrNPV. The low prevalence of parasitism might make it difficult to detect interactions between parasitoids and MabrNPV. The larva of the major parasitoid, a solitary braconid wasp, emerged when the host larva was the size equivalent to the fourth instar. Host larvae survived for a week or longer and in some cases died with a typical symptom of NPV infection and production of OBs, so coexistence of MabrNPV and parasitoid wasps might be possible. Recent reports on interactions between parasitoids and *Spodoptera exigua* MNPV emphasize the possibility to improve the efficacy of biological control against of *S. exigua* with good combinations of the NPV and parasitoids, such that parasitoids have a role as vectors of NPV and contribute to natural epizootics of the virus [56,57].

This two year field study confirmed the enhancing effect of GVP on the MabrNPV infection and potency of the formulations containing MabrNPV at 10^5^ OBs/mL and GVPs at 5 µg/mL for the control of *M. brassicae* larvae. For practical use of MabrNPV, it would be necessary to develop a low cost production system for MabrNPV and XecnGV. This means that we need two independent systems of virus production. Fortunately, MabrNPV and XecnGV are both highly infectious to *Mythimna saparata*, a noctuid pest easily adapted to artificial diets. Even so, it is desirable to develop a new strain of NPV containing the enhancin or synergistic proteins within the occlusion bodies. The method to obtain recombinant baculoviruses in which the foreign proteins were actually incorporated into the occlusion bodies, which retain the ability to occlude virions, was already established [58]. This type of biological insecticide with low impact on the environment may contribute to the expansion of baculovirus use worldwide on the condition the public has a positive perception regarding the benefits of baculovirus GMOs.
